# Scraps

**Published:** 1892-06

**Authors:** 


### SCRAPS

PICKED UP BY THE ASSISTANT-EDITOR.

**Medical Philology** (II.)—In the *Promptorium* will be found the first two of the following quotations. The other two are from the British Museum MS. of the *Catholicon.*

AKE, or ache, or akynge. *Dolor.*
AKYN. *Doleo,* CATH.
To **Ake**; *Noceo, & cetera*; *vbi,* to hurt.
An **Aking**; *Nocumentum.*

The book referred to as CATH, to which the compiler of the *Promptorium* was indebted for the second of the Latin words given, was the *Catholicon* or *Summa* of Johannes de Janua, a dictionary finished by him in 1286. The cross-reference in the third word quoted adds nothing about the word *ake.*

Mr. Herttage's note is:

"'*Doloir.* To grieve, sorrow: to ake, warch, paine, smart.' Cotgrave. Baret points out the distinction in the spelling of the verb and noun: '*Ake* is the Verbe of this substantive *Ache, Ch* being turned into *K.*' Cooper in his *Thesaurus,* 1584, preserves the same distinction. Thus he says—'*Dolor capitis,* a headache; *dolet caput,* my head akes.' The *pt. t.* appears as *oke* in P. Plowman, B. xvii. 194; in Lonelich's *Hist. of the Holy Grail,* ed. Furnivall, and in Robert of Gloucester, 68, 18. A.S. *acan.*"

It is recognised by some of the most recent dictionaries that the present spelling of the verb is wrong. In the *New English Dictionary* it can be seen that the verb is historically *ake,* and was so written even as late as the time of *Dr. Syntax,* 1821. The first extract from the *Promptorium* shows that about 1440 *ake* and *ache* were both used for the spelling of the substantive. That in 1568 it was sometimes pronounced as it is now is clear from a passage in Turner's *Herbal,* in which reference is made to "catarres, runninges of the eyes, and other aykes." But, about that time, as appears from Cooper and Baret, there was a tendency to reserve *ake* for the spelling of the verb and *ache* for that of the substantive, which was pronounced *aitch*. Books of the latter half of the sixteenth century contain many a verbal joke arising out of the identity of sound with that of the letter H. An instance of such pronunciation is found in Henry Chettle's *Kind-Harts Dreame*, 1592, where a description is given of "trauelers that by incilion are able to eafe all aitches" (p. 54 of Reprint in *Shakspere Allusion-Books*, 1874). This pronunciation is clearly referred to by Beatrice (*Much Ado about Nothing*, III. iv. 53–6).

*Beatrice.* By my troth, I am exceeding ill; heigh-ho!
*Margaret.* For a hawk, a horse, or a husband?
*Beatrice.* For the letter that begins them all, H.

Shakspere also makes us familiar with the dissyllabic form of the plural:

Aches contract and starve your supple joints.—*Timon of Athens*, I. i. 257.
Their fears of hostile stroke, their aches, losses.—*Ibid*, V. i. 202.
I 'll rack thee with old cramps,
Fill all thy bones with aches, make thee roar.—*Tempest*, I. ii. 369–70.

This pronunciation continued till the beginning of the 18th century. In "The Description of a City Shower," Swift wrote (*Tatler*, No. 238, October 17, 1710):

A coming shower your shooting corns presage,
Old aches throb, your hollow tooth will rage.

"In the old folio, and first octavo, this word was used as a dissyllable, and so it has continued in all the subsequent editions both of the *Tatler* and Swift's *Works*, till the collection of the English Poets was published in 1779 by Dr. Johnson."—(*Tatler*, ed. 1786, Vol. vi. p. 189). Late editions read "old aches will throb."

In 1806, John Kemble, at Covent Garden, playing Prospero in Dryden and Davenant's version of *The Tempest*, pronounced the word according to the Shaksperian metre, and thereby aroused the anger of the newspaper critics and many regular playgoers. To his credit he did not give way to the ignorant and popular clamour. When it was announced from the stage that one night he was too ill to appear, an unlearned wag cried out, "Mr. Kemble's head *aitches*." During Kemble's absence, the part was played by G. F. Cooke, who, as a way out of the difficulty with which he was confronted, omitted the whole line. A wit then printed this verse as "Cooke's Soliloquy":

"*Aitches* or *akes*, shall I speak both or either?
If *akes* I violate my Shakespeare's measure.
If *aitches* I shall give King Johnny pleasure;
I 've hit upon 't—by Jove, I 'll utter neither."

**Medical Missions.**—Medical missions abroad grow apace, but in very small proportion to the field of work. At the C.M.S. Hospital, Kashmir, there are two European surgeons (Mr. Arthur Neve and his brother, Dr. Ernest Neve), with a qualified house-surgeon and a staff of twelve assistants. These work a hospital with accommodation for upwards of one hundred in-patients, a branch dispensary in the city, and a leper asylum. Visiting is also systematically carried on among all the outlying districts of the valley. During the past year 14,907 new patients were seen and 35,559 visits registered. Such a large proportion of the work is surgical that one patient in five requires operation. Altogether during the year 3,097 operations were performed. Long ranges of wards are filled with the victims of poverty, dirt, hereditary disease, casualties, or contagion. Very few die in the hospital; perhaps in the whole year not more than five or six. Only three of the operation cases died, and one of these was from typhus.

Food is so cheap that the patients get a generous diet. Here is a list of the food supplied on one day to seventy patients: Rice, 135 lbs.; meat, 34 lbs.; milk, 16 pints; sweet oil, 4½ lbs.; salt, 30 ozs.; spices, 27 ozs.; vegetables, about 40 lbs. The total cost of this was about Rs. 6-8, or 10s. 6d.

The work done in the right spirit would be missionary as well as Christian if there were no preaching, for most Christian virtues, notably brotherly kindness, are proclaimed better by deeds than by words. Here is a form of teaching, which none can gainsay, extended in practical benefits to 15,000 people a year, and known by every inhabitant of the valley and the mountains around, and by many in the land of the Great Llama, and in the broad plains of Turkistan and Central Asia. But the doctors aim at being not only the physicians but the friends and spiritual teachers of every patient in the hospital.

The out-patients are seen at mid-day, when they and the whole staff of the hospital are gathered together in the waiting-room. Then one of the lessons for the day is read in Urdu, together with some suitable prayers, followed by an address. In the crowd may be seen the dark faces of the natives from the plains, in contrast with the fair Kashmiris and the ruddy-complexioned Yarkandi; here are the fierce features of the Afghan; there the stalwart form of the Sikh, the wild mountaineer of Chilas, where no European has ever penetrated; the melancholy-looking Gujar, and many another strange tribe of the Himalayas and Central Asia.

Gratitude is not usually a very strong element in the Kashmiri character. The want of it is apt to be rather conspicuous, and although occasionally disagreeable in its manifestations, sometimes it is amusing and sometimes pathetic. An example of a rather gross case of ingratitude was a tailor, who was brought in with a bad strangulated hernia. After operation, he made an uninterrupted recovery. But he was a man who loved a grievance. So instead of rejoicing that he had been snatched from the jaws of death, he made great complaints about being kept in hospital ten days, instead of being allowed to go home at once. More amusing are those cases in which, after recovery from disease, patients are displeased because money is not given to them. An old man, who had been blind in both eyes with cataract, had his sight restored by operation in hospital. His dissatisfaction at not being subsidised quite swallowed up his gratitude for recovery of vision. The pathetic cases are those in which the poor people are so oppressed and down-trodden and miserable that, having found a haven of rest in the hospital, they resent bitterly any attempt to discharge them when cured.

The foregoing has been compiled from various notes written by Mr. Arthur Neve, who came to England recently, and who hopes to deepen home interest in this branch of missionary work. He will doubtless be glad to receive help of any kind sent direct to the hospital or through the Society at its head quarters in Salisbury Square, London, E.C.

Public and professional interest in medical mission work will be further stimulated by the record of the adventures of Dr. Montague, who while at work at a station in British New Guinea was taken prisoner by some native cannibal tribes from Dutch New Guinea. After two and a-half years' captivity he has just escaped.

